# Antiviral Immunity in Amphibians

**DOI:** 10.3390/v3112065

**Published:** 2011-10-31

**Authors:** Guangchun Chen, Jacques Robert

**Affiliations:** Department of Microbiology and Immunology, University of Rochester Medical Center, Rochester, NY 14642, USA; E-Mail: Guangchun_Chen@URMC.Rochester.edu

**Keywords:** iridovirus, viral infection, amphibian

## Abstract

Although a variety of virus species can infect amphibians, diseases caused by ranaviruses ([RVs]; *Iridoviridae*) have become prominent, and are a major concern for biodiversity, agriculture and international trade. The relatively recent and rapid increase in prevalence of RV infections, the wide range of host species infected by RVs, the variability in host resistance among population of the same species and among different developmental stages, all suggest an important involvement of the amphibian immune system. Nevertheless, the roles of the immune system in the etiology of viral diseases in amphibians are still poorly investigated. We review here the current knowledge of antiviral immunity in amphibians, focusing on model species such as the frog *Xenopus* and the salamander (*Ambystoma tigrinum*), and on recent progress in generating tools to better understand how host immune defenses control RV infections, pathogenicity, and transmission.

## 1. Introduction

Amphibians have received much attention during the last two decades because of a now-general understanding that more species are at risk of extinction in this class than those of any other classes of vertebrates [1]. According to the most recent global assessment completed in 2008 [2], nearly one third (32%) of 6,593 amphibian species are threatened with extinction. This number is likely to rapidly increase because many amphibian species with highly restricted ranges are located in those tropical regions where die-offs have occurred. What makes the amphibian case so urgent is that these organisms are long-term survivors that have persisted through the last four mass extinctions in Earth’s history.

While the causes of the global declines of amphibians are multiple and complex (e.g., habitat destruction, introduction of predators/competitors, harmful effects of pesticides or other pollutants, climate change, and increase of ultraviolet-B, *etc.*), infectious diseases now appear to be the proximal causes of death in an important number of cases [3,4]. Among amphibian pathogens the chytrid fungus, *Batrachochytrium dendrobatidis* (*Bd*), is currently the largest infectious disease threat to biodiversity. Because of the impact of wide-spread die-offs all over the world, *Bd* has been directly linked to extinction of amphibian species [5]. Viral infections by Ranaviruses (RV, family *Iridoviridae*) have also become prominent. Although until recently RVs were considered to cause only secondary and limited diseases and dies-off, their prevalence and host-range have recently increased. RVs have become the second most widespread infectious diseases of wild and captive amphibians worldwide. In a recent comprehensive epizootiology study, RVs were identified as the causative agent in approximately half the documented cases of amphibian mortality reported in the United States between 1996 and 2001 [6]. Additional compelling evidence of the worldwide distribution, diversification, and ongoing expansion of RV infections were presented during the First International Symposium on Ranaviruses, Minneapolis MN July 8, 2011 [7–9]. Slides and videos of most presentations are available on the symposium website [10]. Thanks to the momentum initiated by the symposium, a Global Ranavirus Consortium was created to stimulate interactions among ranavirus researchers, and to provide updated information [11]. From these data, it has become clear that RVs have the capability of directly contributing to amphibian population declines. Given the emerging threat of *Bd* and RVs to amphibians, the World Organization for Animal Health [12] now requires the notification of these infectious diseases [13].

RVs are large, icosahedral, double stranded DNA viruses with genomes ranging from 105 to 140 kb that belong to the Iridoviridae family. RVs are capable of infecting three different classes of ectothermic vertebrates: Amphibia, Teleostei, and Reptilia [14], and have fulfilled Koch’s postulates as a causative infectious agent of disease. So far, three RV species infecting amphibians have been identified based on hosts range distributions, nucleotide sequences comparisons, and protein and RFLP profiles [15]. Bohle iridovirus, (BIV), isolated from the native Australian frog, *Limnodynastes ornatus*, remains confined to Australia. *Ambystoma tigrinum* virus (ATV), initially isolated from salamanders in Southern Arizona, infects Ambystomatid salamanders in the US and Canada. In contrast to the relatively limited geographic distribution of these two RV species, frog virus 3 (FV3), the main member and the type species of the RV genus, and originally isolated from the leopard frog *Rana pipiens*, a native North American species, is now found all over the world in a number of different genera and species, potentially making it a serious global threat to amphibians [16].

Despite the goodly amount of data supporting the important role played by RVs, it remains unclear why RVs-associated deaths of amphibian have been noted only recently [15]. In particular, it is currently unclear why some species are susceptible, whereas others are tolerant or even resistant to one or the other RV pathogens. Given the importance of the host immune system in controlling and clearing pathogens, one hypothesis that has been advanced to explain the recent increase in virulence and prevalence resulting in mass die-offs is that at least some amphibian species or populations have abnormally depressed immune systems, perhaps associated with an environmental “stressor” (anthropogenic or otherwise) [17,18]. Therefore, it is urgent to better understand amphibian immune responses to RVs and to identify host genes important for disease resistance, as well as to extend immunological studies to multiple anuran and urodele species. The current review is intended to provide a comprehensive literature on classical and molecular aspects of antiviral responses in amphibians with an indication of the knowledge gaps that are essential to fill in order to institute effective control and prevention of RV infections.

## 2. Organization of the Ectothermic Vertebrate Immune System Compared to Mammals

Host antiviral immune defenses in ectothermic vertebrates and mammals are fundamentally similar, and involve the integration and coordination of two distinct but closely interdependent components: the innate and adaptive immune systems [19,20]. Innate immunity provides a rapid, first line of defense. It includes the production of type I interferon (IFN) by infected cells, which inhibits virus replication by blocking protein synthesis in virus-infected cells and by enhancing natural killer (NK) cell-mediated cytotoxicity [21]. Activation of innate immune responses in vertebrates occurs through the interaction of germ line-encoded pattern recognition receptors (PRRs) on effector cells that recognize molecules specific to pathogens (pathogen-associated molecular patterns [PAMPs]). Engagement of PAMPs by the PRRs initiates biochemical cascades that stimulate effector cells and that induce the release of soluble mediators reacting against different types of pathogens. Innate immunity also includes the release of antimicrobial peptides (AMPs) that are secreted onto the skin from granular glands or by immune cells such as macrophages or neutrophils in the blood and tissues, as well as serum proteins (including acute phase proteins) and complement components that are secreted by the liver [22].

Effector cells of innate immunity can eliminate infected cells by phagocytosis, release of active molecules and cell-mediated cytotoxicity. Among innate effector cells involved in viral immunity are NK cells. These large granulocytic leukocytes play an important role based on their ability to directly kill infected cells and by producing IFN-γ that has antiviral properties and activates other immune cells [23]. In addition, it is known from mammalian studies, that macrophages recruit more phagocytic and effector cells to the area of infection by secreting chemokines such as interleukin-8 (IL-8) and proinflammatory cytokines that include interleukin-1β (IL-1β) and TNF-α [17]. Macrophage also produce active molecules such as reactive oxygen and nitric oxide (NO) that can directly damage the pathogens [24]. We assume that similar cytokines and other active molecules are released in amphibians.

While the innate system responds rapidly, the adaptive immune system may take several days to become fully activated, and requires prior exposure to an antigen to mount a full immunological response, utilizing both cell-mediated and humoral responses. Adaptive immune responses are characterized by B and T cells expressing a huge variety of clonal surface Ag-specific receptors, which in contrast to germline-encoded innate PRRs, are somatically generated by recombination-activating genes (RAG)-dependent gene rearrangements to detect the pathogens and provide the host an immunological memory [19]. The vertebrate adaptive immune system is evolutionarily more recent than innate immune systems. It appeared near the time of the emergence of jawed vertebrates ∼500 million years ago (MYA) [20]. The adaptive immune response starts by the expansion of the antigen-specific T cell clones and their differentiation into effectors. Macrophages are also implicated in adaptive immune responses as professional antigen presenting cells (APCs) that can process viral antigens through Major Histocompatibility Complex (MHC) class I and class II presentation pathways that then activate CD8 and CD4 T cell effectors, respectively [25]. Other APCs that are more efficient than macrophages are multiple subsets of dendritic and Langerhans cells. These APCs are well studied in mammals but are still poorly defined in ectothermic vertebrates [20]. CD8 T cells give rise to cytotoxic T cells (CTLs) that can kill virally-infected cells by recognizing viral antigen peptide complexed with MHC class I at their surface. CTLs produce also large amount of IFN-γ and other cytokines (e.g., TNF-α). In mammals, CD4 T cells differentiate into various T helper effectors (Th1, Th2, Treg, *etc*.) that produce cytokines important for the production of CTLs (e.g., IL-2) and B cells. Although function of mammalian T helper-like cells (e.g., mixed lymphocyte reaction) and many of the genes specifying the different CD4 T cell subsets have been identified in bony fish and amphibians, the presence and function of these subsets in ectothermic vertebrates is still unclear. The second arm of the adaptive immune response is constituted by B cells that differentiate into plasma cells and produce antibodies that can directly neutralize the virus or promote antibody-dependent cell-mediated cytotoxicity. The peak of the adaptive response usually leads to the clearance of the virus and is followed by a contraction phase during which most of the T cell effectors are eliminated by programmed cell death, except a minor fraction of memory T cells that can survive for a long time and can respond faster to a second infection. There are also memory B cells and long-lived antibody-secreting plasma cells. The relative importance and interaction of the different immune cells vary depending of the virus considered and is still the subject of active research by numerous scientists.

## 3. The *Xenopus* Immune System

From an evolutionary point of view, *Xenopus* is one “connecting” taxon that links mammals to vertebrates of more ancient origin (bony and cartilaginous fishes) that shared a common ancestor ∼350 MYA [20]. Importantly, *Xenopus* is a “transitional” animal model, being the oldest vertebrate class in which the immunoglobulin (Ig) class switch occurs, but does so in the absence of germinal center formation critical for T cell-dependent B-cell maturation in mammals. In addition to its wide use for developmental studies, *Xenopus* has been, and still is frequently used as the nonmammalian comparative model of choice for comparative immunological studies. Most of the fundamental knowledge about the immune system in amphibians comes from the extensive studies in *X. laevis*, which provided the foundation for the analysis of *S. tropicalis* genomic sequences, and allowed identification of many immunologically-relevant gene homologs. The *Xenopus* immune system has recently been the object of a comprehensive review [20]; here we provide just a succinct summary.

Studies with *X. laevis* over several decades have revealed the fundamental conservation of the immune system and its high degree of similarity to the mammalian immune system [26]. NK cells and most other typical leukocyte types such as neutrophils, basophils, eosinophils, polymorphonuclear cells, monocyte and macrophage-like cells, and smaller lymphocytes can be observed in the blood and the peritoneal fluid. Although *Xenopus* lacks the mammalian equivalent of lymph nodes and a lymphopoietic bone marrow, it does have a thymus where T cells differentiate and a spleen that represents the main peripheral lymphoid organs where both B and T cells accumulate in the white pulp, especially in the follicular area where IgM^+^ B cell surrounded by T cells aggregate around a central blood vessel [27]. Lymphocytes and other leukocytes also accumulate in the periphery of the liver, the kidneys, and along the intestine but without forming the organized lymph nodes as in mammals. In contrast to mammalian mature B cells that are generally not phagocytic, peripheral differentiated B cells from teleost fish species and *X. laevis* are phagocytic and capable of killing ingested microbes [28]. This finding suggests that evolutionarily, B cells and macrophages may share a common origin.

At the gene level, many of the gene homologs involved in mammalian innate immunity have been identified in *X. laevis* and *S. tropicalis* [20]. Among them, Toll-like receptors (TLR) are one of the innate receptors that recognize PAMPs on pathogens that initiate innate as well as adaptive immune responses. Of interest, in contrast to mammals that have 10 TLRs, a total of 20 different TLR genes, as well as some adaptor proteins, have been identified in the *S. tropicalis* genome [29,30]. All these TLR genes are constitutively expressed in tadpoles and adults, suggesting that the innate immune response through TLR signaling is active throughout life. While most TLRs are evolutionarily conserved due to the strong selection for maintenance of specific PAMP recognition, *Xenopus* TLR4 (*i.e.*, the receptor responsible of response to the endotoxin lipopolysaccharide [LPS] in mammals; reviewed in [22]) seems to be divergent. In this regard, it is interesting to note that *Xenopus* is poorly responsive to purified LPS (e.g., adult can receive up to 1 mg of LPS without any sign of inflammation or other untoward effects) [31]. Thus, *Xenopus* carries all the human orthologs and some TLR family members that are expanded in a *Xenopus*-specific manner (e.g., TLR14).

As in mammals, the development and function of the adaptive immune system depend on MHC molecules. MHC class I-restricted cytotoxic and MHC class II-restricted helper T cell responses have been identified in *X. laevis*. Most of the molecules that define adaptive immunity (e.g., Igs, T-cell receptor [TCR], MHC, RAG, activation-induced cytidine deaminase [AID]) have been characterized [20]. Although putative dendritic cells and Langerhans cells have been described in *Xenopus* adult skin based on morphological criteria and some markers such as MHC class II Ag, and vimentin [32], it is not yet known if these cells present antigens. However, APC activity of peritoneal macrophages has been characterized [33]. The somatic repertoire of TCRs and Ig receptors are generated in a RAG-dependent manner, and B cells produce antibodies of IgM, IgD, IgY (IgG-equivalent) and IgX isotypes [34]. IgY is the functional equivalent of mammalian IgG isotype, and the thymus dependency of the switch from IgM to IgY is consistent with T helper function [20]. It is noteworthy that despite these fundamental similarities of the immune systems of *X. laevis* and mammals, affinity maturation in *Xenopus* is poor when compared with mammals. For example, the affinity of *X. laevis* IgY antibody against dinitrophenol (DNP), a model antigen, increases less than 10 times during a humoral response in contrast to more than a 10,000 fold affinity increase in mammals [35,36].

A unique feature of *X. laevis* that is likely shared by all anuran species is the presence of distinct immune systems in the two developmental life stages, larval and adults, as well as the dramatic changes occurring during the metamorphosis. For examples, although both *Xenopus* larvae and adults are immunocompetent and have CD8 T cells, larvae lack significant expression of MHC class I until metamorphosis [37]. This strongly suggests an absence of class I-restricted T cell education during larval life. Presumably related to the suboptimal expression of MHC class I, NK cells are not detected until late in larval stage at the time of metamorphosis [38]. Furthermore, cell-mediated cytotoxicity involving either CTLs or NK cells cannot be detected in larvae and becomes significant only several weeks after metamorphosis is completed [38]. The relative weakness of the larval adaptive immune system extends to antibody production and T helper function, since the switch from IgM to IgY antibodies of higher affinity is poor in larvae [34,35]. Therefore, the existing data strongly suggest that the larva displays weak adaptive cell effectors, and thought to rely critically on its innate immune system.

## 4. Immunity to RVs in *Xenopus* and Other Anuran Species

*Xenopus* provides a powerful experimental model to study immunity to RV diseases since not only is its immune system the most extensively characterized of any amphibian, but MHC-defined strains and clones as well as a large panel of monoclonal antibodies (and established assays to use them) are available [39]. Moreover, at least under laboratory conditions, *Xeno*pus is susceptible to FV3, and tadpoles seem less able to defend themselves. The high susceptibility of larval stage to RV infection is also documented for other anuran species in natural and captive population. Therefore, comparison in *Xenopus* between susceptible tadpoles and resistant adults to RV infection provides ways to elucidate virulence and immune escape mechanisms that are of significant fundamental relevance.

### 4.1. Adults

**Figure 1.**
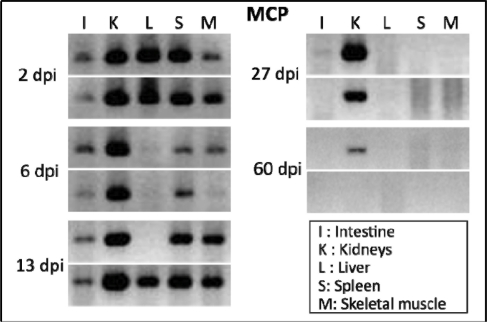
Slow clearance of FV3 DNA in *X. tropicalis*. FV3 DNA detected by PCR (35 cycles) using primers specific for the major capsid protein (MCP) on genomic DNA purified from various tissues of outbred ***X****. tropicalis* adults that were infected with FV3 by i.p. injection of 1 × 10^6^ PFU for 2, 6, 13, 27 and 60 days (2 individuals per time point).

Initial study revealed that FV3 infection of adult *X. laevis* is pathogenic (∼10−20% of adults infected with 10^7^ pfu die within a month) [39]. Infected frogs that died exhibit both edema and hemorrhages. Frogs that survive the FV3 infection show only transitory signs of pathology (e.g., lethargy, loss of appetite, cutaneous erythema of the legs, skin shedding). These symptoms disappear within a few weeks. Similar symptoms and resistance were also observed using ATV and *Rana catesbeiana* virus Z (RCV-Z), a FV3-like virus [40]. Interestingly, whereas viral DNA is detected by PCR in most tissues of infected moribund frogs, the kidney is the primary target of FV3 in *X. laevis*. Immunohistology of tissues from infected frogs using an anti-FV3 monoclonal antibody has confirmed that the *X. laevis* kidney is the primary target of FV3 [41]. Extensive necrosis of proximal tubules in parallel with accumulation of detectable viruses is typically observed during early stages of infection. We have observed similar resolution of symptoms of infection in a preliminary study with FV3 in *X. tropicalis*. Like *X. laevis*, the kidney appears to be the main tissue infected in *X. tropicalis*. However, the virus clearance in this organ is considerably slower than it is in *X. laevis.* (e.g., viral DNA still detected four weeks post-infection; [40]). In fact, viral DNA has been detected in a few asymptomatic animals 2 months post FV3 infection (Figure 1). This suggests that as in the case of *X. laevis*, quiescence phase of FV3 infection can also occur in *X. tropicalis*. Despite their overall similar morphology, *X. laevis* and *X. tropicalis* belong to distinct evolutionary lineages whose common ancestor dates back 60 MYA. The possible conservation of covert infection by FV3 in these two species could provide a powerful comparative system of investigation.

**Figure 2.**
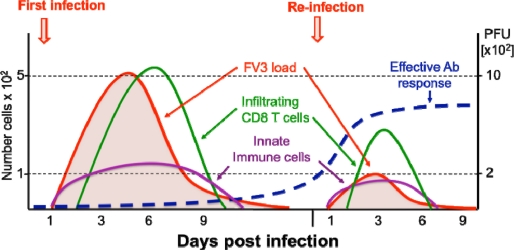
Schematic view of *Xenopus* adult immune response kinetics in infected kidneys. During both primary and secondary FV3 infections, MHC class II+ innate immune cell effectors (leukocytes) rapidly accumulate in the kidneys (violet line), the main site of infection, and pro-inflammatory genes (e.g., TNF-α, IL-1β) are induced. This is followed by an adaptive CD8 T cell response and infiltration (green line) that peak at 6 dpi during a primary infection. During a second FV3 infection, CD8 T cell response and infiltration peak 3 days earlier, which suggests T cell memory. However, the lower number of infiltrated CD8 T cells (5 time less) suggests that anti-FV3 antibodies (blue line) and B cell memory are playing a prominent role during secondary infection resulting in a faster viral clearance (red line).

The kinetics of viral clearance in adult *X. laevis*, as measured by loss of FV3 DNA, correlates with onset of T cell and B cell responses that peak at 6 dpi. Both sub-lethal γ-irradiation-induced thymocyte depletion and monoclonal antibody depletion of CD8 T cells markedly increase the susceptibility of adults to FV3 infection, indicating the crucial role of CTLs in *X. laevis* in controlling FV3 infection [42]. We have further developed a flow cytometry assay using bromodeoxyuridine (BrdU) incorporation to assess lymphocyte proliferative responses *in vivo*, and have detected significant proliferation of splenic CD8 T cells 6 days after FV3 infection. Tissue infiltration of activated CD8 T cells was monitored by immunohistology. Following primary infection, CD8 T cells significantly proliferate in the spleen and accumulate in infected kidneys from day 6 onward in parallel with virus clearance (Figure 2). Earlier proliferation and infiltration associated with faster viral clearance were observed during a secondary FV3 infection [42]. However, there was a decrease of CD8 T cells proliferating in the spleen and infiltrating in the kidneys compared to the primary response. Therefore, although these results provide evidence of a protective CD8 T cell response in *X. laevis* against FV3 as well as the occurrence of CD8 T cell memory, they also suggest the involvement of other effector mechanisms during a re-infection. For example, it is possible that a more potent antibody response becomes prominent during a secondary infection (see below), as is the case for poxvirus [43]. In any case, these results provide evidence that amphibians like *Xenopus* can develop protective Ag-dependent CD8 T cell proliferation, recognition, and memory against a natural viral pathogen.

It is important to mention that so far no specific anti-FV3 IgM or IgY Abs have been detected by ELISA in the sera of frogs for up to a month after they were infected for the first time with FV3. However, increased mRNA expression of IgY and AID, an enzyme essential for the maturation, indicates that B cells are activated during primary FV3 infection [27]. More studies are needed to determine if a primary FV3 infection induces antibodies at a too low titer or at a too low affinity to be detected by our assay. Nonetheless, specific anti-FV3 IgY Abs are detected after a second viral infection (2 to up to 6 months after initial exposure; Figure 2), and viral clearance is markedly accelerated (*i.e.*, no viral DNA detectable 3 days post-infection), indicating that protective antibodies are generated following a secondary infection [44]. Therefore, when examined in a physiological context involving a natural viral pathogen, antibodies generated by *X. laevis* do appear to provide protective defenses against subsequent viral infection even though these antibodies are of a weaker affinity than their mammalian counterpart.

Notably, specific antibodies against RVs have been detected in the serum of the marine toad *Bufo marinus* from Australia and Venezuela [45]. Also a prior exposure of bullfrogs (*Rana catesbeiana*) to FV3 (relatively avirulent in this species) protects against a subsequent challenge with RCV-Z, a more virulent FV-3-like virus strain [46]. All these observations are consistent with immunological CD8 T cell and B cell memory, which means that as in mammals, the adaptive immune system of adult frogs provides a faster and more potent protection against a second RV infection. Taken together, these results strongly suggest that the clearance of RVs in amphibians involves the host’s adaptive immune system.

Compared to adaptive immunity, much less is known about the role of innate immunity in FV3 infection. As a first step, we have investigated (using microscopy, flow cytometry and RT-PCR) the contribution of peritoneal leukocytes (PLs) in the immune response to FV3 by adult *X. laevis* [33]. Besides the active involvement of NK cells during early stages of FV3 infection (*i.e.*, before the onset of T cell responses), our study reveals that macrophages are also involved. The total number and the relative abundance of macrophages rapidly increases from 1 to 6 days post-infection, and these cells display an activated morphology including phagocytic vacuoles. FV3 infection also induces a rapid up-regulation of pro-inflammatory genes including Arginase 1, IL-1β and TNF-α that are likely to be produced in large part by macrophages.

Although almost nothing is known about innate immune response to RVs in anuran species other than *X. laevis*, leukocyte accumulations at the site of infection of *Rana temporaria* reported on necropsies are consistent with the involvement of innate cell effectors [6,47]. Interestingly, several antimicrobial peptides produced at the surface of the skin of *Rana pipiens* and *Rana catesbeiana* inactivate FV3 *in vitro*, which suggests that these compounds can contribute to innate defenses against RV infection [48,49]. As is likely with other facets of innate immunity, AMPs may play an important role in inactivating viruses at their portals of entry and controlling infections prior to the onset of adaptive immune responses. Moreover, if skin and mucus membrane concentrations of AMPs are adversely affected by environmental conditions, then lower levels of these peptides may predispose amphibian populations to serious disease.

### 4.2. Larvae

In contrast to adults, *Xenopus* larvae are considerably more susceptible to FV3, showing more than 80% morbidity over 2 months [39]. It is presumed that this reflects immature and/or less efficient adaptive effector functions. These include a lack of MHC class I protein expression, which, in mammals, is necessary for CTL responses, and antibodies of lower affinity than adult due to the poor switch of IgM to IgY. However, the variability of survival times observed between individuals suggests that the larval immune system is not completely inactive or ignorant of FV3 infection. Furthermore, although *Xenopus* tadpoles do not express class I until metamorphosis, they do have CD8 T cells. Whether these cells function immunologically in larvae and are class I unrestricted or restricted by nonclassical MHC class I are an interesting research area.

Several other studies are also consistent with a higher susceptibility of larvae and metamorphs to RVs. Unlike *Rana pipiens* adults that survive infection by injection of 10^6^ pfu of FV3, embryos and tadpoles succumb to injections of doses as small as 900 pfu [50]. Several RV reported outbreaks appear to preferentially affect tadpoles. The massive death reported in ranaculture, the practice of farm-raising bullfrogs (*R. catesbeiana*) for scientific and culinary purposes, also mainly targets tadpoles and individuals that have just metamorphosed [51,52].

So far, our attempts to detect any type of larval *Xenopus* anti-FV3 immune response have had limited success. We have found that as in adults during primary infection, IgY and AID mRNA expression is up-regulated in larval B cells. More recently, we developed highly sensitive immunoprecipitation and western blotting techniques to detect anti-FV3 IgY antibodies. Preliminary results with FV3-immunized tadpoles show that some specific signals can be detected. In addition, young adults that were primed and survived FV3 infection at the larval stage developed a typical anti-FV3 IgY secondary response upon re-infection [40]. Moreover, prior infection of bullfrog tadpoles with relatively avirulent FV3 protects against subsequent challenge with RCV-Z, a more virulent FV-3-like virus strain [46]. This suggests that the primary FV3 infection in larvae has generated a long lasting thymus-dependent B cell memory, which has persisted through metamorphosis. This would imply that tadpoles surviving FV3 infection may become more resistant to a secondary infection at the adult stage.

Besides the observed absence of NK cells until metamorphosis, little is known about innate immune responses in larvae. To explore this area we recently investigated the response kinetics of several innate immune genes during the early phase of FV3 infection. Using quantitative real-time PCR, we found only a modest (10–100 times lower than adults) and delayed (3 days later than adults) up-regulation of TNF-α, IL-1 and IFN-γ genes in leukocytes and in infected tissues, as well as a delayed induced expression of the type I IFN-inducible Myxovirus-resistance (Mx) 1 gene. Our study suggests that the immaturity of the larval immune system extends to innate effector components, which further weaken larval immune defense to RV infections [53]. Immune responses of *Xenopus* adults and larvae are summarized in Table 1.

### 4.3. Complex Role of Xenopus Macrophages in Host Defenses and Viral Persistence

**Figure 3.**
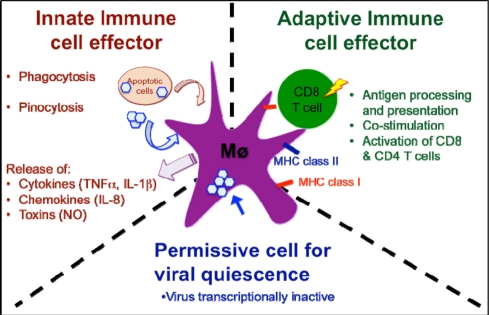
Schematic view of the complex role of macrophages in *Xenopus* host defenses against RV. As innate immune cell effectors, macrophage can acquire viral antigen by direct infection, pinocytosis of opsonized viruses or phagocytosis of infected cells, as well as release cytokines, chemokines and toxins that contribute to limiting the infection. As an adaptive immune cell effector, macrophages process and present viral antigens through MHC class I and class II pathways, up-regulated co-stimulatory molecules (B7, CD40) and activate anti-RV CD8 and CD4 T cells. Finally, macrophage can harbor quiescent RV in asymptomatic frogs.

As mentioned before, in *Xenopus* as in mammals, macrophages are key cell effectors in both innate and adaptive immunity (Figure 3). Of particular interest with regard to viral persistence, our study provides evidence of the particular permissiveness of certain PLs to FV3 infection [33]. Notably, the persistence of transcriptionally inactive FV3 genomic DNA in PLs may explain the occurrence of asymptomatic infection and suggests that FV3 is capable of covert infection. Although some PLs are susceptible to FV3 infection as evidenced by apoptotic cells, active FV3 transcription and the detection of viral particles by electron microscopy, the infection is weaker (fewer infectious particles), more transitory and involves a lower fraction (less than 1%) of PLs than the kidney, the main site of infection. However, viral DNA remains detectable in PLs for at least 4 weeks post-infection; this is past the point of viral clearance observed in the kidneys.

**Figure 4.**
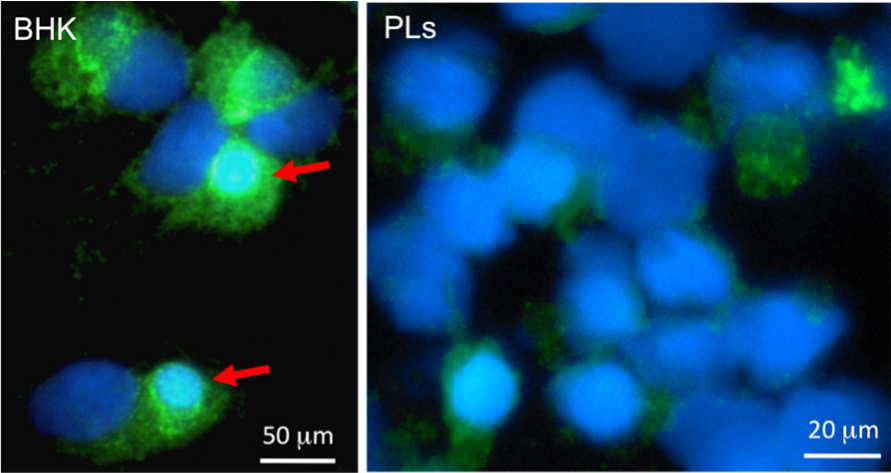
Immunofluorescence microscopy of baby hamster kidney cells (BHK, left) and *Xenopus* PLs infected *in vitro* for 2 days with FV3 (0.3 MOI). Cells were cytocentrifuged on microscope slides, fixed with formaldehyde, permeabilized with ethanol, incubated with a rabbit anti-53R and FITC-conjugated donkey anti-rabbit Abs (Green); then stained with the DNA dye Hoechst-33258 (Blue) mounted in anti-fade medium and visualized with a Leica DMIRB inverted fluorescence microscope. Note the large viral assembly sites in BHK cells that contain large amount of viral DNA stained Hoechst-33258 and anti-53R Ab (arrows). In contrast, anti-53R staining is weaker in PLs, and no assembly sites are detected.

Recently, we have developed a multicolor immunofluorescence method to characterize macrophage infected by FV3: PLs infected with FV3 *in vitro* for 2 days were double stained with mouse anti-HAM56, which recognizes the macrophage antigen HAM56 and specifically cross-reacts with *Xenopus* macrophages [54], and rabbit anti-53R, kindly provided by Dr. V.G. Chinchar that recognizes ORF 53R, a putative 54.7-kDa myristoylated viral protein that is critical for FV3 replication [55]. A clear co-localization of the viral 53R antigen and the macrophage specific HAM56 was observed in a consistent fraction of PLs, which provides direct and clear evidence of macrophage infection by FV3 [56]. Notably, FV3 infectivity is lower in PLs than in BHK-21 cells: anti-53R staining is weaker, and no assembly sites are detected (Figure 4).

Taken together these results suggest that although PLs are actively involved in anti-FV3 immune responses, some of these cells can be permissive and harbor quiescent, asymptomatic FV3. It is currently unknown how common and relevant is the ability of FV3 or other RVs to establish transient quiescent infections in their hosts and what are the mechanisms involved. However, subclinical infections of several species have been documented, which is consistent with a quiescent phase of RV infection [8,57,58].

### 4.4. MHC Genotype and Susceptibility to RVs

In *X. laevis* there is a single MHC class I gene per genome. Our preliminary comparison among *X. laevis* outbred (putatively MHC heterozygous), and the J and F inbred strains that are MHC homozygous for different haplotypes suggests a higher susceptibility of the J homozygous genotype [39]. J homozygous adults required twice as long (2-months) to clear the infection compared to heterozygous outbreds. The susceptibility of the J strain was even more apparent in tadpoles; 100% morbidity occurred within 2 weeks following FV3 infection compared to 80% within 2 months for outbred tadpoles. Interestingly, J strain adults seem to have a lower level of MHC class I surface expression than other strains or clones examined thus far [59]. This strain could provide an important tool for further investigation of a possible association between MHC genotype and host susceptibility to FV3 infection.

The evidence from a natural experiment with *Rana temporaria* in the field where some ponds have been exposed to repeated RV infections for over a decade, whereas others have been free of disease over the same period of time, suggest that certain MHC supertypes (*i.e.*, a group of MHC molecules that are able to bind overlapping set of peptides with common motifs) are associated with infection status (even after accounting for shared ancestry), and the diseased populations have more similar supertype frequencies than the uninfected, implying directional selection against the alleles conferring greater susceptibility [60]. This finding not only provides genetic evidence for the important involvement of the adaptive immune system in anti-FV3, but also indicates that, in the wild, frogs may be able to adapt over time to the presence of RVs.

## 5. The Immunity to RVs in Salamanders

Relative to other vertebrate models, the axolotl immune response has often been described as immunodeficient [61]. There are several reasons for this characterization, including: lack of white and red pulp compartmentalization of their spleen [62], the production of only two Ig classes, only one of which regulates the humoral response and neither of which is anamnestic; no detectable humoral response to soluble antigens [63]; antibody titers to horse or sheep erythrocytes reaching a peak only two months after priming [64]; chronic rejection of skin allografts, poor mixed lymphocyte reactions and relatively weak *in vitro* proliferative responses to T- and B-cell mitogens, and lack of cellular cooperation during the humoral immune response as indicated by enhanced humoral immunity following thymectomy or X-ray irradiation [61,65]. In addition, axolotl has an expanded MHC class I repertoire (∼100 genes) and a non-polymorphic MHC class II [66]. Based primarily on the characteristically chronic rejection of allografts and xenografts, weak immune responses appear to extend to many other species and genera of salamanders [67].

The weakness of the salamander’s immune system is well illustrated by RV infection. Tiger salamanders (*Ambystoma tigrinum*) are highly susceptible to ATV infections with high mortality rates both in the laboratory and in the field. In contrast to FV3 infection, where mortality is more common in larvae than adults, both larval and adult salamanders succumb to ATV infection, and mortality in affected ponds often exceeds 90% [15]. Symptoms are typical of RV infection: lethargy, slow movement, red spots or swollen areas near the gills and hind limbs. Hemorrhages and ulceration of the skin, edema, swollen and pale livers, and fluid-filled intestines are also seen. Temperature influences the extent of mortality and time to death as most salamanders infected at 26 °C survive, whereas lower temperatures (10 °C) at which immune responses are likely to be inefficient, result in mortality of almost all infected animals [68]. Experimental attempts to determine the host range of ATV demonstrated that various salamander species (*Ambystoma graciale*, *A. californiense*, *Notophthalmus viridescens*) are susceptible to infection, but bullfrogs (*R. catesbeiana*) and fish (*Gambusia affinis*, *Lepomis cyanellus*, *Oncorhynchus mykiss*) are resistant to infection [15]. Attempts to determine whether antimicrobial peptides are involved in the immune response of tiger salamanders to ATV revealed inconsistent effects [69]. Whereas some natural mixtures of peptides from tiger salamanders reduced ATV-induced viral plaques, not all preparations of skin peptides were equally effective. However, some evidences of innate immune responses in axolotls (*Ambystoma mexicanum*) has been obtained using microarray technology [61]. These limited studies of the *Ambystoma* defense responses to ATV should be considered in the context of the finding that *in vivo* and *in vitro* immune responsiveness of urodeles are noticeably less “robust” than those of anurans. *Ambystoma mexicanum* infected with ATV appears to mount some innate immune response. However, gene expression changes indicative of lymphocyte proliferation in the spleen, which is associated with clearance of FV3 in adult *Xenopus was* not detected. Therefore, it has been speculated that ATV may be especially lethal to *A. mexicanum* and related tiger salamanders because they lack proliferative lymphocyte responses that are needed to clear highly virulent RVs. However, more direct evidence of lack of lymphocyte proliferation during ATV infection (e.g., BrdU incorporation, *etc.*) is needed before any definitive conclusion can be drawn.

## 6. Viral Immune Evasion and Virulence Proteins

Understanding the precise roles that innate and adaptive responses play in anti-ranaviral immunity is challenging, but the observation that viruses encode proteins that inhibit specific immune pathways provides “biological proof” for the importance of those responses in host defense. Moreover, because host antiviral defense mechanisms and virus-encoded virulence genes likely represent different sides of the same coin, elucidating key elements of the anti-viral response can benefit from a detailed characterization of viral gene functions.

Virulence/immune evasion genes are genes that facilitate the virus to replication *in vivo* (and in some cases *in vitro*) by impairing host antiviral responses. Based on analogy with poxviruses and herpesviruses, several potential immuno-escape genes have been identified by bioinformatics using fully sequenced genome of several RVs [70]. In FV3, candidate immune evasion/virulence genes include a viral homolog of the α subunit of eukaryotic initiation factor eIF-2 (vIF-2α), a CARD (caspase recruitment domain-containing protein, vCARD), an hydroxysteroid dehydrogenase homolog (vHSD), a viral homolog of the tumor necrosis factor receptor (vTNFR), a ribonuclease III (RNase)-like protein, a cytosine DNA methyltransferase (DMT), and several ORFs encoding putative proteins containing immunoglobulin- or MHC-like domains. Moreover, since the aforementioned genes were identified by amino acid sequence identity/similarity with their well-characterized mammalian counterparts, it is likely that other, less conserved, immune evasion proteins remain to be discovered. Last but not least, two-thirds of these putative gene products share no sequence similarity with known viral or eukaryotic proteins, and therefore are of unknown function. Clearly, characterizing the precise function of these putative virulence/immune evasion gene homologs and identifying potential new RV-specific virulence/immune evasion are important for better understanding the success of RV pathogens including their capacity to adapt and expand its host and geographic ranges. To reveal functions of putative virulence/immune evasion genes in RVs we have recently developed an improved technique to knockout specific genes by homologous recombination [71]. The implementation of this improved method to generate FV3 knockout mutants provides a powerful way not only to identify viral genes involved in virulence and immune evasion but also to develop an attenuated viral vaccine. We have already targeted two FV3 genes: a truncated *eIF-2α* and the immediate early gene *18K*.

The eIF-2α protein subunit is involved in translational control and host interferon downregulation in eukaryotic cells. Phosphorylation of eIF-2α leads to the shutdown of the translational machinery of the host, which prevents viral replication and therefore is an efficacious method of antiviral defense. Viruses must inhibit elevated levels of phosphorylated eIF-2α within the cell to successfully replicate. Like poxviruses, most RVs encode vIF-2α, which acts as a pseudosubstrate that binds the antiviral protein kinase PKR and prevents the phosphorylation and subsequent inactivation of cellular eIF-2α [72]. Consistent with this view, knockout of vIF-2α from ATV leads to increasing pathogenicity and sensitivity to interferon [73]. Interestingly, FV3, like shell turtle iridovirus (STIV), only encodes an N-terminal truncated version of vIF-2α, which is not able to block PKR activity, and, therefore, was assumed to be non-functional [74]. Moreover, FV3 is less virulent for tadpoles than RCV-Z that possesses a full-length vIF-2α. To better assess the roles of FV3 genes in virulence and immune evasion, we knocked out putative virulence vIF-2α by homologous recombination and assessed their roles in replication *in vitro* and *in vivo*. Unexpectedly, our observation indicate that the truncated vIF-2α is still critically involved in viral growth and virulence in *Xenopus* tadpoles, since the challenge experiments showed that survival of the tadpoles infected with vIF-2α-KO FV3 was significantly increased [71]. These results suggest that some feature(s) within this truncated molecule contributes to virulence. Although we have not been able to detect a difference in the host immune response, more detailed studies of the expression of immune-related genes will be needed to reveal subtle differences in antiviral immunity. This study bridges the disciplines of virology and immunology since elucidating the identity and function of virus-encoded immune evasion molecules will likely identify specific elements of the host immune response that play critical roles in antiviral immunity. The *18K* gene encodes an abundant immediate early RV-specific protein of yet unknown function. The recombinant FV3 18K KO mutant showed impaired virulence and growth upon infection in *Xenopus* tadpoles, thereby providing the first evidence that this gene is involved in RV virulence [71].

## 7. Role of Host Immune System in Pathogenesis Transmission and Persistence of RV Infections

Although there is still little direct evidence, host resistance to viral infection may be affected by environmental factors, such as temperature, pollutants, habitat modification and invasive species. Some of these factors may weaken host immune function directly or indirectly (e.g., stress-related), and therefore increase the success of pathogens by enhancing their growth or virulence, as well as their dissemination [17,18]. The possible significance of impaired amphibian immune function and resulting increased disease susceptibility in global amphibian declines is supported by the survival of some amphibian species despite the introduction of pathogens. For example, not all amphibian species are susceptible to pathogens of global concern. The American bullfrogs (*R. catesbeiana*) and *X. laevis* carry *Bd* infections without significant signs of chytridiomycosis [18]. These findings suggest that specific immune defenses are required for protection against pathogens and play a key role in population success, rather than success depending on the pathogen's virulence alone. Some populations of the same species, more susceptible to RV than others, may be due to different exposure to pollutants and/or different stress.

Among climate related factors, exposure to cold temperature has been reported to impair some immune functions of *R. pipiens* (e.g., lower level complement in the serum, mitogen-induced T cell proliferation), although it did not alter host resistance to infection with the bacteria *Aeromonas hydrophila* [75]. Ultraviolet-B radiation (UVB) can be immunosuppressive and has been reported to increase amphibian susceptibility to infection, although this is likely to be through a complex and unclear combination with other factors [76,77]. Many studies have documented the immunosuppressive effects of pollutants such as pesticides, suggesting that environmental contaminants may play a role in increased pathogen virulence and disease rates [18]. Laboratory exposure to agricultural pesticides can result in inhibitory effects on immune functions of amphibians, such as decreased peripheral leukocyte levels, altered spleen cellularity, impaired lymphocyte proliferation responses, and compromised skin peptide defenses [18]. Furthermore, chronic exposure to atrazine, a potent endocrine disruptor in amphibians, not only interferes with metamorphosis of amphibians, but also alters expression of genes involved in immune function and development [78]. The concentration used is this study (400 parts per billion) is environmentally relevant since the Environmental Protection Agency’s “Recommended Water Quality Criterion” for atrazine is 350 parts per billion. Furthermore, atrazine and several other pesticides also alter cellularity and phagocytic activity of *X. laevis* and lymphocyte proliferation of *R. pipiens* [79]. Exposure to insecticide also increases mortality of *A. tigrinum* to ATV infection [80]. However, it is important to note that there are some perplexing instances in which environmental factors is not clearly linked to population declines, particularly in relatively undisturbed areas, such as wilderness areas of the western United States, rain forests of Central America and pristine areas in Australia. In most of these cases, infectious diseases seem to be the direct cause of die-offs in amphibians [17].

As an alternative way to investigate the relevance of immune status in viral susceptibility and dissemination, we developed a cross-infection model in which the adults of *X. laevis* were immunocompromised by sub-lethal γ-irradiation [56]. Our data showed that immunocompromised adults as well as immature tadpoles are susceptible to FV3 infection by waterborne transmission from both immunocompromised and immunocompetent infected adults with whom they are cohoused. At the individual level, impairment of immunity is likely to modify pathogenesis. In *X. laevis*, we have shown that FV3 localization in immunocompetent adults is mostly limited to kidneys, and that FV3 remains localized to discrete area that are rapidly cleared by the immune response. By contrast, in immuncompromised adults, the infecting virus becomes rapidly systemic and spreads to other organs like the liver and intestine, and is accompanied by hemorrhages. Such infected animals release infectious particles in the water that can infect other interspecific and conspecific animals including tadpoles. Although the available data emphasize adaptive immunity, defective components of the innate immune system may also contribute to increase host susceptibility to RV infection by weakening or delaying the initiation of the adaptive immune response. In this scenario, disease outbreaks and decline of amphibian populations associated with RVs are an indirect effect of environmental pressures (*i.e.*, “stressors”) on the host immune system.

Another relevant issue for discussion concerns the potential ability of RV to take advantage of the host immune system to persist and increase its dissemination. The viral genes potentially involved in immune evasion mentioned at the end of the previous section give a first hint of this possibility. Asymptomatic feral adults of different species including *X. laevis* have been reported to carry RVs. RV infection in captive adult anurans may occur without clinical signs or consistent histopathologic lesions. In addition, our study in *X. laevis* has shown that FV3 can infect macrophages and remain transcriptionally inactive for up to 3 weeks in these cells. This suggests that FV3 is capable of covert infection as are some other iridoviruses in insect [81]. In turn, this type of infection may contribute to the dissemination of the disease. Asymptomatic carriers can serve as a viral reservoir that under immunocompromising conditions develops a systemic infection that rapidly spreads in a population that is subjected to the same immunocompromising conditions.

Although the susceptibility of various developmental stages of salamanders to RV may differ from anurans, data obtained so far are also consistent with a tight dependence of efficient immunity and resistance to RVs. Larval salamanders become infectious soon after exposure to ATV. Interestingly, atrazine exposure also increases susceptibility to ATV infection in larvae and decreases peripheral leukocyte counts in adults [82]. This is an indication that environmental contaminants may have immunosuppressive effects on tiger salamanders to ATV infection as it may in anuran species. A recent survey of ATV in natural salamander population indicates a relatively high prevalence of the virus in animals not associated with morbidity [83]. Therefore, it is possible that ATV may also be able of covert infection in salamanders.

## 8. Concluding Remarks

As discussed extensively in this review, there is now compelling evidence for mass deaths among amphibian populations resulting from infectious disease outbreaks. Although amphibians have effective and diverse immune defense mechanisms, the failure of these defenses to prevent RV infection suggests that environmental factors may be compromising the status of their immune system. Owing to the increased threat of emerging wildlife viral diseases on global biodiversity, more fundamental and comparative research on viral immunity is needed. Extensive studies of amphibian immunity have become a key issue if one wants to understand how these viruses can persist, disseminate and expand their host ranges. A major challenge in studying antiviral immunity (especially adaptive immune responses) in cold blooded vertebrates is the absence of species-specific tools (*i.e.*, antibodies and primers specific for immunologically-relevant gene products) and MHC-matched host systems. The use of appropriate animal models such as *Xenopus* and *Ambystoma* is a critically important first step in examining viral-host interactions. However, future studies will need to include other amphibian species. New methodologies for global analysis of transcriptomes such as high-throughput deep sequencing, combined with the established effective approaches of knock down and knock out for RVs, should help unravel complex antiviral mechanisms in amphibians and the strategies employed by viruses to avert the immune responses. As the immune systems of ectothermic vertebrates become better understood, it is likely that their roles in protecting fish, amphibians, and reptiles from RVs infections will become clearer and be utilized to prevent or predict RVs infections.

## Figures and Tables

**Table 1.** Summary of immune responses to FV3 in larval and adult *X. laevis*.

	**Adults**		

	**Primary**	**Secondary**	**Larvae**

**Symptoms**	2–3 weeks	3–5 days	Long lasting, >80% death
**Virus Clearance**	1 month	1 week	Ineffective

***Innate Immunity***			
**Cells**	1 dpi: Activated Mø	Same + Mø as APC Similar to primary	Mø less resistant to FV3 No NK
	3 dpi: NK cells 1		
**Induced genes**	dpi: TNF-α, IL-1β, IFN-γ, Mx1	Similar to primary	Delayed (3 dpi) and weaker

***Adaptive T cell immunity***			
**Splenic CD8 T cell**	Peak at 6 dpi	Peak at 3 dpi but lower expansion	?
**CD8 T cell in kidneys**	At 6 dpi	At 3 dpi but fewer	?
**T cell memory**	-	yes	?

***Adaptive B cell immunity***			
**Anti-FV3 antibodies**	Not detected	IgY from 10 dpi	Not detected
**More IgY mRNAs**	6 dpi	3dpi	6–7 dpi
**AID up-regulation**	9 dpi	3 dpi	6–7 dpi
**B cell memory**	-	Yes	Possibly

Abbreviations: AID: activation-induced cytidine deaminase; dpi: days post-infection; Mø: Macrophages.
